# A Western Dietary Pattern Is Associated with Poor Academic Performance in Australian Adolescents

**DOI:** 10.3390/nu7042961

**Published:** 2015-04-17

**Authors:** Anett Nyaradi, Jianghong Li, Siobhan Hickling, Jonathan K. Foster, Angela Jacques, Gina L. Ambrosini, Wendy H. Oddy

**Affiliations:** 1 School of Population Health, The University of Western Australia, Perth 6009, Australia; E-Mails: Siobhan.Hickling@uwa.edu.au (S.H.); Gina.Ambrosini@uwa.edu.au (G.L.A.); Angela.Jacques@uwa.edu.au (A.J.); 2 Telethon Kids Institute, The University of Western Australia, Perth 6008, Australia; E-Mails: jianghong.li@wzb.eu (J.L.); J.Foster@curtin.edu.au (J.K.F.); Wendy.Oddy@telethonkids.org.au (W.H.O.); 3 WZB Berlin Social Research Center, Reichpietschufer 50 D-10785 Berlin, Germany; 4 Centre for Population Health Research, The Faculty of Health Sciences, Curtin University, Perth 6102, Australia; 5 School of Psychology & Speech Pathology, Curtin University; Perth 6102, Australia; 6 Neurosciences Unit, Health Department of Western Australia; Perth 6010, Australia; 7 School of Paediatrics & Child Health, The University of Western Australia, Perth 6008, Australia

**Keywords:** diet, academic performance, adolescence, Raine Study

## Abstract

The aim of this study was to investigate cross-sectional associations between dietary patterns and academic performance among 14-year-old adolescents. Study participants were from the Western Australian Pregnancy Cohort (Raine) Study. A food frequency questionnaire was administered when the adolescents were 14 years old, and from the dietary data, a ‘Healthy’ and a ‘Western’ dietary pattern were identified by factor analysis. The Western Australian Literacy and Numeracy Assessment (WALNA) results from grade nine (age 14) were linked to the Raine Study data by The Western Australian Data Linkage Branch. Associations between the dietary patterns and the WALNA (mathematics, reading and writing scores) were assessed using multivariate linear regression models adjusting for family and socioeconomic characteristics. Complete data on dietary patterns, academic performance and covariates were available for individuals across the different analyses as follows: *n* = 779 for mathematics, *n* = 741 for reading and *n* = 470 for writing. Following adjustment, significant negative associations between the ‘Western’ dietary pattern and test scores for mathematics (β = −13.14; 95% CI: −24.57; −1.76); *p* = 0.024) and reading (β = −19.16; 95% CI: −29.85; −8.47; *p* ≤ 0.001) were observed. A similar trend was found with respect to writing (β = −17.28; 95% CI: −35.74; 1.18; *p* = 0.066). ANOVA showed significant trends in estimated means of academic scores across quartiles for both the Western and Healthy patterns. Higher scores for the ‘Western’ dietary pattern are associated with poorer academic performance in adolescence.

## 1. Introduction

Adolescence is a period of life when major psychosocial and biological changes occur, resulting in the highest nutrient requirement at any time across the lifecycle [1]. Adolescence is also an important stage for brain development, characterized by synaptic pruning, myelination and a growing number of neural connections, especially in the prefrontal cortex [2,3]. Adolescence is a vulnerable period of the life course with regard to nutrition because, with increasing independence from parents, food choices are more frequently made by adolescents. During this time of development, peer pressure and media promotion exert a relatively greater influence on food purchases, often in favour of less healthy nutritional choices [1].

The academic performance of children and adolescents has been a focus for public health researchers. School performance influences future education, which ultimately shapes an individuals’ socioeconomic status; in which in turn, is associated with health and health behaviour [4]. Nutrition is one of the most important and modifiable environmental factors that may affect brain development, and therefore cognition and academic performance [5].

Relatively little research has reported the effect of diet on academic performance in adolescents. Having regular lunch and dinner were negatively associated, while higher consumption of soft drinks, pizza, hot dogs, sweets and snacks that indicated poor diet were positively associated with self-reported learning difficulties in mathematics in Norwegian adolescents. Moreover, regular breakfast in the same study was negatively linked with learning difficulties in mathematics, reading and writing [6]. In Korea, regular meals have been linked with higher academic performance in adolescents [7]. A study in Iceland reported lower academic achievement in adolescents with increased consumption of French fries, hamburgers and hot dogs indicating poor dietary habits and higher academic scores in adolescents with positive dietary habits, consuming more fruits and vegetables [8]. In Sweden, adolescents who consumed more fish during the week, had higher academic grades [9]. Researchers showed positive associations between higher fruits, vegetables and milk consumption and academic achievement in Canadian adolescents [10].

In the present study, based on the findings reported in the extant literature, we hypothesised that a healthier diet would be associated with higher academic achievement (specifically mathematics, reading and writing achievement) and conversely a western type diet with poorer academic performance in Australian adolescents at 14 years of age.

## 2. Methods

### 2.1. Study Population

The study utilised data from the Western Australian Pregnancy Cohort (Raine) Study. In the original study, 2900 pregnant women from Perth, Western Australia, were serially recruited between 1989 and 1991 into a randomised controlled trial to study the effects of pregnancy ultrasounds on the newborn [11]. The women, enrolled in the study at between 16 to 20 weeks’ pregnant when they presented at King Edward Memorial Hospital, the major tertiary maternity facility in Perth, Western Australia and surrounding private practices. A total of 2868 babies (born between 1989 and 1992) and their families were followed up at regular intervals. Ethics approval was granted by The Human Ethics Committee at King Edward Memorial Hospital and Princess Margaret Hospital for children. The current study was approved by the University of Western Australia Human Research Ethics Committee.

Access to the educational data through a data linkage process was approved by the Western Australian Department of Health Human Research Ethics Committee. Parents and participants provided informed consent at each follow-up. At aged 18 years Raine Study participants consented to future follow-up investigations. For this analysis, we included data collected at the one, eight and 14-year cohort follow-ups (*i.e.*, core data collected at age 14 plus data for potential confounders collected at earlier time points).

### 2.2. Dietary Patterns

Dietary data were collected using a semi-quantitative food frequency questionnaire (FFQ) developed by the Commonwealth Scientific and Industrial Research Organisation (CSIRO), Adelaide, Australia [12,13]. This questionnaire was administered and evaluated at 14 years of age in the Raine cohort [14]. The FFQ was mailed to study families; primary caregiver completed the FFQ, in consultation with the adolescent participant and 1631 questionnaires were returned for analysis. Information on the usual frequency of consumption and typical serve size over the past 12 months for 212 foods was collected by the FFQ. Intakes of these foods were then grouped into 38 major food groups, measured as grams per day of intake [15].

Dietary patterns were derived by factor analysis (sample size = 1613) from the major food groups; this process was limited to factors with an eigenvalue >1, and varimax rotation was undertaken to improve the separation and interpretability of the factors [15]. Two major dietary patterns were identified where factor 1 explained 50% of the common variance shared by food group intakes (13% of total variance) and factor 2 explained 34% of the common variance in food intakes (8.5% of the total variance); these patterns were named ‘Healthy’ (factor 1: high in fruits, vegetables, whole grains, legumes and fish) and ‘Western’ (factor 2: high intake of take-away foods, red and processed meat, soft drinks, fried and refined food) [15]. Each subject received a dietary pattern score, measured as a *z*-score for each pattern (one dietary pattern does not exclude the other pattern in an individual because a combination of foods are eaten). Total energy intake was estimated by linking the recorded food intakes for each individual from the FFQ with the Australian Food Composition Tables by the CSIRO [15]. Total energy intake was included in our analysis as a covariate. Details of the methodology, the reliability and the validity of the FFQ have been previously published [14,15,16]. The dietary patterns with factor loadings are shown in Supplementary Table 1.

### 2.3. Western Australian Literacy and Numeracy Assessment (WALNA) Score

The Western Australian Literacy and Numeracy Assessment (WALNA) was administered and collected by the Western Australian Department of Education to all students in Western Australia annually in grades three (age eight years), five (age ten years), seven (age 12 years) and nine (age 14 years) between 1998–2007. This was part of an Australia-wide program, such that the findings are comparable with similar assessment programs undertaken in other Australian states and their results were reported against nationally agreed benchmarks. The WALNA data include test results for mathematics, reading, writing (in grades three, five, seven and nine) and spelling (in grades three, five and seven). These educational data were obtained from a combination of multiple-choice, open-response and short-response questions, and only year nine data are reported here. The standardized raw scores for each of the mathematics, reading, writing and spelling scores were summed via an ordinal scale and converted into an interval scale. This process was completed using a Rasch measurement model [17] for easier understanding and interpretation of results. In the Rasch score all subjects are placed on a common scale, and it is a standard practice in the analysis of educational data as shown in previous studies [18,19].

With respect to the interval scale for all four areas of assessment (mathematics, reading, writing and spelling), higher scores represent higher levels of achievement. Educational professionals assessed the content and construct validity of the WALNA measures each year; these analyses demonstrate an internal reliability of 0.8 [18].

In our study, we used the grade nine (age 14 years) WALNA data that included mathematics, reading and writing scores. A probabilistic method of matching at the individual level (based on a full name, date of birth, gender and address) was used by The Western Australian Data Linkage Branch to link the WALNA to the Raine study has an accuracy of >99% (18). Once the links were created, only deidentified data from both sources were provided to the researchers for analysis, ensuring that no individual level data were accessed as part of the ‘separation principle’ [20].

### 2.4. Covariates

Sociodemographic characteristics identified as maternal education, maternal race, family income, family functioning and the presence of the biological father in the family were included in the analyses as potential confounders. Maternal education (collected at the eight year follow-up in the Raine Study) was considered in eight categories: (i) did not finish high school; (ii) finished high school and completed the tertiary entrance exam; (iii) trade/apprentice certificate; (iv) college/TAFE (Technical and Further Education) certificate; (v) diploma; (vi) bachelor degree; (vii) postgraduate degree; and (viii) ‘other’. Maternal race was characterized into three categories (Caucasian; Aboriginal; “other”), while family income (collected at the 14 year follow-up) was classified according to four levels: ≤$25,000; $25,001–$50,000; $50,001–$78,000; and >$78,000 per annum. Family functioning (14 years follow-up) was included in the analysis as a continuous variable (higher scores represented better functioning) and was measured by the McMaster Family Assessment Device [21]. This measure collected information about family communication, problem solving, affective responsiveness and behaviour control. The presence of the biological father in the family when the child was 14 years of age was dichotomised as ‘yes’ or ‘no’.

Characteristics of adolescents including gender, body mass index ((BMI) (kg/m^2^) weights and heights) and the level of physical activity (outside of school hours) were obtained. We included these variables in the statistical models, because both BMI and physical activity have been associated with cognitive performance and academic achievement in adolescents [22,23]. BMI was grouped into four categories defined by Cole [24]: underweight, normal weight, overweight and obese. Participants were assigned to three categories of physical activity using a questionnaire, as per previous studies using Raine data [15]: <1 time/week; 1–3 times/week; and 4+ times/week.

The diet score at one year of age was based on the infants’ dietary intake over the previous 24 h. Data from more than 2000 foods were collapsed into food groups and a continuous score was developed that provided a score between 0–70 (higher score representing better diet) [25]. This diet score was included in the analyses, as it was previously found in the Raine Study that diet during infancy was associated with cognitive development in middle childhood and may be a predictor for later academic performance [26,27].

### 2.5. Statistical Analysis

The data were initially analysed to generate descriptive statistics. We then built three models using multivariable linear regression to evaluate the relationship between diet and the WALNA scores at age 14. In model one, we minimally adjusted for total energy intake to ensure that outcomes were independent of total energy consumption. In model two, we additionally adjusted for maternal education and race, family income and functioning, the presence of biological father in the family, diet score at one year and child gender. In model three, we adjusted for all variables that had been included in models one and two, but additionally included adolescent BMI and physical activity level to determine if these covariates modified our results. Finally, we examined 21 key food groups identified as the main contributors to the dietary patterns (as continuous variables, measured by grams/day intake) with factor loadings ≥0.30 across both ‘Healthy’ and ‘Western’ dietary patterns (Table 1) in order to identify those food groups specifically associated with academic scores. All analyses were performed using IBM SPSS Statistics 22. Results are reported using a significance level (*i.e.*, alpha) of 0.05.

## 3. Results

Table 1 lists the characteristics of the Raine cohort included in this study. Complete data for mathematics, reading and writing scores were available for *n* = 779, *n* = 741 and *n* = 470 adolescents, respectively. The descriptive statistics did not differ significantly across all three samples according to the academic subjects.

**Table 1:** Descriptive characteristics of the Western Australian Pregnancy Cohort (Raine) Study at age 14 by educational outcomes, mathematics, reading and writing at 14 years.

Continuous Variables	Sample 1—Mathematics *n* = 779	Sample 2—Reading *n* = 741	Sample 3—Writing *n* = 470
	Mean (SD)	Mean (SD)	Mean (SD)
Mathematics (grade nine)	541.14 (86.75)		
Reading (grade nine)		497.75 (78.46)	
Writing (grade nine)			574.77 (104.91)
Healthy dietary pattern	−0.08 (0.88)	−0.07 (0.88)	−0.09 (0.80)
Western dietary pattern	−0.07 (0.81)	−0.08 (0.81)	−0.07 (0.74)
Total energy intake (KJ)	9298.89(2792.38)	9267.44 (2790.26)	8949.42 (2541.27)
Diet quality score (age one follow-up)	42.52 (9.97)	42.67 (9.83)	42.84 (9.80)
Family functioning score	1.79 (0.44)	1.79 (0.45)	1.79 (0.45)
**Categorical Variables**	***n* (%)**	***n* (%)**	***n* (%)**
Maternal education (age eight follow-up)			
not finished high school	199 (25.5)	183 (24.7)	112 (23.8)
finished high school, tertiary entry exam	138 (17.7)	134 (18.1)	80 (17.0)
trade/apprentice certificate	26 (3.3)	22 (3.0)	15 (3.2)
collage/TAFE certificate	157 (20.2)	146 (19.7)	90 (19.1)
diploma	92 (11.8)	92 (12.4)	58 (12.3)
bachelor degree	83 (10.7)	83 (11.2)	59 (12.6)
postgraduate degree	57 (7.3)	56 (7.5)	42 (9.0)
other	27 (3.5)	25 (3.4)	14 (3.0)
Maternal race			
Caucasian	719 (92.3)	683 (92.2)	433 (92.1)
Aboriginal	6 (0.8)	6 (0.8)	3 (0.6)
other (*i.e*., Asian)	54 (6.9)	52 (7.0)	34 (7.3)
Family income			
≤AUS$25,000	94 (12.1)	87 (11.7)	54 ((11.5)
AUS$25,001–AUS$50,000	240 (30.8)	228 (30.8)	117 (24.9)
AUS$50,001–AUS$78,000	217 (27.9)	207 (27.9)	133 (28.3)
>AUS$78,000 per annum	228 (29.2)	219 (29.6)	166 (35.3)
Father presence in the family			
yes	498 (63.9)	481 (64.9)	316 (67.2)
no	281 (36.1)	260 (35.1)	154 (32.8)
BMI			
normal	544 (69.8)	518 (69.9)	325 (69.2)
underweight	46 (5.9)	47 (6.3)	31 (6.6)
overweight	129 (16.6)	120 (16.2)	73 (15.5)
obese	60 (7.7)	56 (7.6)	41 (8.7)
Physical activity			
≥4 times per week	279 (35.8)	266 (35.9)	187 (39.8)
1–3 times per week	416 (53.4)	396 (53.4)	228 (48.5)
<1 time per week	84 (10.8)	79 (10.7)	55 (11.7)
Gender of the child			
female	390 (50.1)	372 (50.2)	238 (50.6)
male	389 (49.9)	369 (49.8)	232 (49.4)

Table 2 shows the results of the multivariate linear regression models for each academic performance score in relation to dietary patterns (both as continuous variables and quartiles). In model one, one standard deviation higher *z*-score for the ‘Western’ dietary pattern (continuous variable) at 14 years of age was associated with lower test scores for mathematics (β = −29.05; 95% CI: −39.50; −18.61; *p* ≤ 0.001), reading (β = −26.47; 95% CI: −6.00; −16.93; *p* ≤ 0.001) and writing (β = −27.71; 95% CI: −44.00; −11.43; *p* = 0.001). Further, a one standard deviation higher *z*-score on the ‘Healthy’ dietary pattern (continuous variable) was associated with higher scores in mathematics (β = 9.28; 95% CI: 2.83; 15.72; *p* = 0.005), reading (β = 12.74; 95% CI: 6.84; 18.64; *p* ≤ 0.001) and writing (β =18.87; 95% CI: 8.12; 29.62; *p* = 0.001).

In model two, these results remained significant with respect to the ‘Western’ dietary pattern (continuous variable) (mathematics (β = −14.95; 95% CI: −25.87; −4.04; *p* = 0.007), reading (β =−19.38; 95% CI: −29.53; −9.23; *p* ≤ 0.001) and writing (β = −18.16; 95% CI: −35.51; −0.82; *p* = 0.040)), but were no longer significant for the ‘Healthy’ dietary pattern (continuous variable).

With respect to model three, the associations with the Western dietary pattern (continuous variable) were not altered by BMI and physical activity for mathematics (β = −13.14; 95% CI: −24.57; −1.76); *p* = 0.024) or reading (β = −19.16; 95% CI: −29.85; −8.47; *p* ≤ 0.001). However, the association with writing scores was attenuated from −18.16 (β = −17.28; 95% CI: −35.74; 1.18; *p* = 0.066). This difference in outcome for writing between model two and model three may be due to a Type II statistical error due to the lower sample size for the writing scores (*n* = 470) compared with the mathematics (*n* = 779) and reading (*n* = 741) scores. Higher BMI was associated with a lower mathematics score (F = 3.81, *p* = 0.010) in model three, but there were no associations between BMI and reading or writing. Physical activity was not associated with any of the WALNA scores in model three. The final model explained 19%–20% of variance (adjusted R squared) in academic performance. More detail concerning the associations between ‘Western’ and ‘Healthy’ dietary patterns (as continuous variables) and mathematics, reading and writing scores and covariates at age 14 are provided in Table 3. When dividing the ‘Healthy’ and ‘Western’ dietary patterns into quartiles, the results of the multivariate linear regression models were similar to the previously described associations between the continuous dietary pattern scores and academic outcomes (results are presented in Table 2).

Table 4 presents the estimated adjusted means for mathematics, reading and writing scores for the quartiles of ‘Western’ and ‘Healthy’ dietary pattern scores (estimated according to the predicted values derived from the fitted models). There was an estimated 46 point decrease in mathematics score, 59 point decrease in reading score and 57 point decrease in writing score, comparing adolescents in the first quartile of the ‘Western’ dietary pattern (the lowest level) to the fourth quartile (highest level) and 9 points increase in mathematics, 28 points increase in reading and 42 points increase in writing scores when comparing the ‘Healthy’ dietary pattern first and fourth quartiles. Although ANOVA for trend was significant for both the ‘Western’ and ‘Healthy’ dietary patterns regarding the estimated means of academic outcome scores, the multivariate regression analysis did not show significant associations between the ‘Healthy’ pattern and academic outcomes after adjusting for the covariates.

**Table 2:** Multivariate regression models between WALNA scores at grade nine (age 14) and dietary patterns (both as continuous variables and as quartiles) at age 14 in the Western Australian Pregnancy Cohort (Raine) Study.

WALNA Scores	Dietary Patterns	Model 1 **		Model 2 **		Model 3 ****	
(Grade Nine)	(Continuous and Quartiles *)	β (95% CI)	*p*	β (95% CI)	*p*	β (95% CI)	*p*
**Mathematics**	Healthy	9.28 (2.83; 15.72)	0.005	3.14 (−3.68; 9.97)	0.366	4.37 (−2.78; 11.51)	0.231
*n* = 779	Western	−29.05 (−39.50; −18.61)	0.001	−14.95 (−25.87; −4.04)	0.007	−13.14 (−24.57; −1.76)	0.024
**Reading**	Healthy	12.74 (6.84; 18.64)	0.001	3.88 (−2.42; 10.17)	0.227	5.47 (−1.15; 12.09)	0.105
*n* = 741	Western	−26.47 (−36.00; −16.93)	0.001	−19.38 (−29.53; −9.23)	0.001	−19.16 (−29.85; −8.47)	0.001
**Writing**	Healthy	18.87 (8.12; 29.62)	0.001	3.67 (−8.06; 15.41)	0.539	4.84 (−7.57; 17.25)	0.444
*n* = 470	Western	−27.71 (−44.00; −11.43)	0.001	−18.16 (−35.51; −0.82)	0.040	−17.28 (−35.74; 1.18)	0.066
**Mathematics** *n* = 779	Healthy						
	4st Quartile	28.72 (12.90; 44.54)	<0.001	8.39 (−8.75; 25.52)	0.337	12.25 (−5.74; 30.24)	0.182
	3nd Quartile	9.90 (−5.49; 25.29)	0.207	3.35 (−12.91; 19.62)	0.686	5.63 (−11.52; 22.77)	0.520
	2rd Quartile	4.21 (−11.42; 19.841)	0.597	2.18 (−13.95; 18.31)	0.791	4.69 (−12.36; 21.75)	0.589
	1th Quartile	0		0		0	
	Western						
	4st Quartile	−50.77 (−72.28; −29.25);	<0.001	−23.24 (−45.79; −0.69)	0.043	−22.40 (−45.62; 0.82)	0.059
	3nd Quartile	−30.41 (−49.31; −11.52)	0.002	−16.80 (−36.30; 2.70)	0.091	−17.83 (−37.94; 2.28)	0.082
	2rd Quartile	−13.49 (−29.88; 2.91)	0.107	−5.62 (−22.42; 11.19)	0.512	−5.25 (−12.36; 21.75)	0.558
	1th Quartile	0		0		0	
**Reading** *n* = 741	Healthy						
	4st Quartile	37.20 (22.59; 51.82)	<0.001	13.86 (−2.14; 29.86)	0.089	17.93 (0.95; 34.90)	0.038
	3nd Quartile	21.20 (7.07; 35.34)	0.003	6.68 (−8.48; 21.84)	0.387	8.83 (−7.27; 24.93)	0.282
	2rd Quartile	19.51 (5.17; 33.86)	0.008	13.79 (−1.22; 28.81)	0.072	14.04 (−1.96; 30.03)	0.085
	1th Quartile	0		0		0	
	Western						
	4st Quartile	−45.11 (−64.95; −25.27)	0.000	−29.52 (−50.60; −8.44)	0.006	−30.45 (−52.34; −8.57)	0.006
	3nd Quartile	−28.68 (−46.10; −11.26)	0.001	−21.92 (−40.07; −13.77)	0.018	−20.36 (−39.26; −1.45)	0.035
	2rd Quartile	−13.76 (−28.90; 1.39)	0.075	−15.05 (−30.83; 0.73)	0.062	−13.56 (−30.28; 3.17)	0.112
	1th Quartile	0		0		0	
**Writing** *n* = 470	Healthy						
	4st Quartile	47.43 (22.33; 72.53)	0.000	15.77 (−11.91; 43.45)	0.264	21.96 (−7.17; 51.09)	0.139
	3nd Quartile	22.50 (−0.59; 45.58)	0.056	5.85 (−19.38; 31.08)	0.649	11.85 (−14.77; 38.46)	0.382
	2rd Quartile	19.63 (−4.07; 43.33)	0.104	18.91 (−6.33; 44.16)	0.142	20.51 (−6.20; 47.22)	0.132
	1th Quartile	0		0		0	
	Western						
	4st Quartile	−50.64 (−84.10; −17.17)	0.003	−31.20 (−67.02; 4.62)	0.088	−29.90 (−67.28; 7.48)	0.117
	3nd Quartile	−31.64 (−61.13; −2.16)	0.035	−21.86 (−67.02; 4.62)	0.165	−20.59 (−52.94; 11.75)	0.212
	2rd Quartile	−8.63 (−34.64; 17.39)	0.515	1.68 (−25.79; 29.14)	0.904	2.12 (−27.48; 31.72)	0.888
	1th Quartile	0		0		0	

* 1st quartile = lowest level; 4th quartile = highest level. ** Model 1 includes: Healthy and Western dietary pattern, total energy intake. *** Model 2 includes: all variables in model 1 plus maternal education, maternal race, family income, the presence of biological father in the family, family functioning, diet quality at age one and gender. **** Model 3 includes: all variables in model 2 plus BMI and physical activity.

**Table 3:** Detailed multivariate regression analysis associations between Western and Healthy dietary patterns and mathematics, reading and writing scores and covariates at age 14 in the Western Australian Pregnancy Cohort (Raine) Study.

	Mathematics		Reading		Writing	
	β (95% CI)	*p*	β (95% CI)	*p*	β (95% CI)	*p*
Healthy dietary pattern	4.37 (−2.78; 11.51)	0.231	5.47 (−1.15; 12.09)	0.105	4.84 (−7.57; 17.25)	0.444
Western dietary pattern	−13.14 (−24.57; −1.76)	0.024	−19.16 (−29.85; −8.47)	0.001	−17.28 (−35.74; 1.18)	0.066
Total energy intake	−0.002 (−0.005; 0.002)	0.386	−0.001 (−0.004; 0.003)	0.684	0 (−0.006; 0.006)	0.985
Diet quality score (age one follow-up)	0.59 (−0.002; 1.18)	0.051	0.27 (−0.29; 0.83)	0.348	0.74 (−0.19; 1.67)	0.117
Family functioning	−6.99 (−19.72; 5.75)	0.282	−8.71 (−20.51; 3.09)	0.148	−10.57 (−30.37; 9.22)	0.294
Maternal education (age eight follow-up)						
not finished high school	−36.95 (−68.75; −5.16)	0.023	−30.52 (−60.70; −034)	0.047	−02.09 (−55.40; 51.21)	0.938
finished high school, tertiary entry exam	−22.67 (−55.18; 9.84)	0.171	−26.50 (−57.12; 4.12)	0.090	−5.57 (−60.06; 48.91)	0.841
trade/apprentice certificate	−20.28 (−62.54; 21.97)	0.346	4.80 (−36.19; 45.79)	0.818	29.07 (−40.54; 98.67)	0.412
collage/TAFE certificate	−22.34 (−54.43; 9.75)	0.172	−18.11 (−48.53; 12.31)	0.243	−6.13 (−60.10; 47.84)	0.824
diploma	4.76 (−28.96; 38.49)	0.782	−14.85 (−46.54; 16.83)	0.358	29.45 (−26.38; 85.28)	0.300
bachelor degree	3.46 (−30.77); 37.70)	0.843	1.51 (−30.67; 33.68)	0.927	28.52 (−27.82; 84.86)	0.320
postgraduate degree	45.29 (9.03; 81.56)	0.014	26.80 (−7.33; 60.92)	0.124	64.14 (5.49; 122.79)	0.032
other	0		0		0	
Maternal race						
Caucasian	−39.51 (−61.52; −17.51)	<0.001	−23.06 (−43.50; −2.63)	0.027	−55.95 (−89.83; −22.06)	0.001
Aborigines	4.44 (−62.12; 70.99)	0.896	−23.51 (−84.40; 37.39)	0.449	−11.30 (−124.74; 102.13)	0.845
Other (*i.e*., Asian)	0		0		0	
Family income						
≤AUS$25,000	−33.50 (−55.49; −11.61)	0.003	−28.95 (−49.49; −8.42)	0.006	−52.52 (−87.31; −17.79)	0.003
AUS$25,001–AUS$50,000	−20.04 (−35.90; −4.19)	0.013	−19.49 (−34.27; −4.70	0.010	−27.26 (−52.98; −1.53)	0.038
AUS$50,001–AUS$78,000	−11.18 (−26.33; 3.97)	0.148	−2.42 (−16.57; 11.74)	0.737	−2.33 (−25.34; 20.69)	0.843
>AUS$78,000 per annum	0		0		0	
Father presence in the family		0.211		0.456		0.620
no	−8.50 (−21.85; 4.84)		−4.74 (−17.21; 7.74)		5.51 (−16.29; 27.30)	
yes	0		0		0	
BMI						
normal	26.60 (5.19; 48.01)	0.015	14.47 (−5.67; 34.60)	0.159	16.85 (−15.80; 49.50)	0.311
underweight	37.05 (6.36; 67.73)	0.018	17.78 (−10.39; 45.94)	0.216	20.90 (024.96; 66.75)	0.371
overweight	8.02 (−16.19; 32.23)	0.516	4.33 (−18.47; 27.13)	0.709	−4.17 (−41.28; 32.94)	0.825
obese	0		0		0	
Physical activity						
≥4 times per week	−0.97 (−20.83; 18.89)	0.924	2.26 (−16.34; 20.87)	0.811	7.22 (−22.54; 36.98)	0.634
1–3 times per week	−4.31 (−22.90; 14.28)	0.649	4.02 (−13.47; 21.51)	0.652	4.39 (−24.12; 32.90)	0.762
<1 time per week	0		0		0	
Gender of the child		0.004		0.002		<0.001
male	17.80 (5.81; 29.79)		−17.38 (−28.59; −6.17)		−46.40 (−65.43; 027.31)	
female	0		0		0	

**Table 4:** Estimated means (from predicted values of multivariable regression models) of academic scores for the quartiles of Western and Healthy dietary patterns scores in the Western Australian Pregnancy Cohort (Raine) Study at 14 years of age.

Estimated Mean for the Whole Sample with SD	Estimated Mean Mathematics Score *	Estimated Mean Reading Score *	Estimated Mean Writing Score *
	541.14 (41.42)	497.75 (36.52)	574.77 (50.87)
**Healthy dietary pattern**			
1st quartiles	524.99	480.94	551.22
2nd quartiles	540.55	498.72	574.68
3rd quartiles	548.94	505.22	583.79
4th quartiles	554.44 **	509.61 **	593.18 **
**Western dietary pattern**			
1st quartiles	562.35	525.49	601.79
2nd quartiles	544.86	504.56	576.99
3rd quartiles	536.67	487.65	560.26
4th quartiles	516.30 **	466.15 **	545.04 **

* Adjusted for Healthy and Western dietary patterns, total energy intake, maternal education, maternal race, family income, the presence of the biological father in the family, family functioning, diet quality at one year, gender, BMI and physical activity. ** *p* Values for trend in ANOVAs are all <0.001.

**Figure 1:**
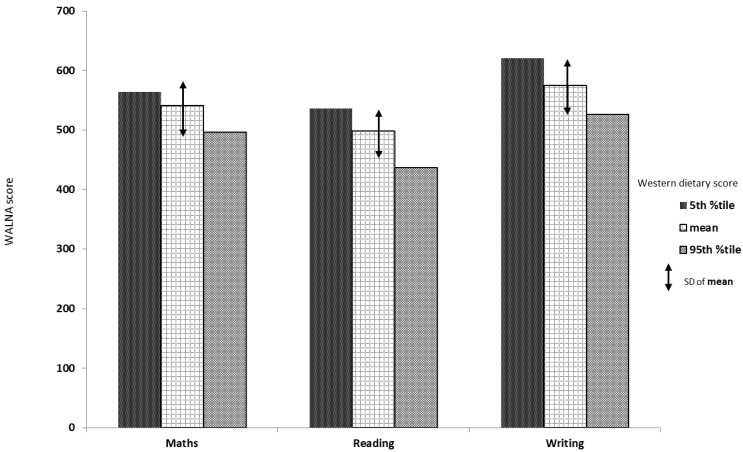
The differences in the predicted adjusted academic scores between the 5th percentile (lowest level) and 95th percentile (highest level) of the Western dietary pattern score in the Western Australian Pregnancy Cohort (Raine) Study at 14 years of age.

Further, we examined the difference in the predicted adjusted academic scores between adolescents in the 5th percentile (lowest level) and 95th percentile (highest level) of the ‘Western’ dietary pattern score. We found that the estimated mean mathematics score for the 5th percentile of the ‘Western’ dietary pattern score was 563.64 compared with the whole sample (mean 541.14; SD = 41.42), with a difference of 22.5 points (*i.e.*, 0.54 SD above the sample estimate), while for the 95th percentile the mean mathematics score was 495.91 (*i.e*., 45.23 points (1.09 SD) below the whole sample mean). Similarly, the estimated mean reading score at the 5th percentile was 536.18 compared with the whole sample (mean 497.75; SD = 36.52) with a difference of 38.43 points (1.05 SD above the sample estimate), while at the 95th percentile the estimated mean was 436.44 with a difference of 61.31 points (1.68 SD below the sample mean). The estimated mean writing score at the 5th percentile was 620.49, which was 45.72 points (0.90 SD) above the whole sample mean (574.77; SD = 50.87); the mean writing score for the 95th percentile was 525.62, which was 49.15 points (0.97 SD) below the whole sample mean. Figure 1 illustrates these findings.

**Figure 2:**
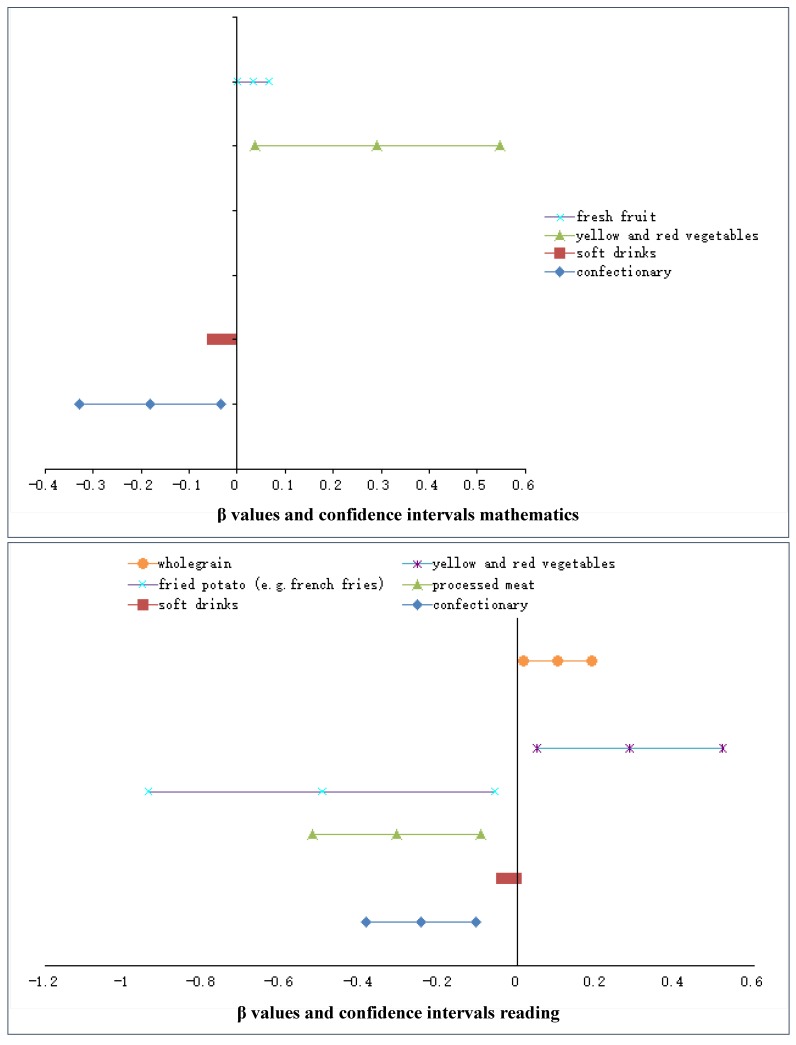
Significant associations in multivariable regression models between food groups and academic outcomes (mathematics and reading) in the Raine Study illustrated in forest plots by β values and 95% confidence intervals.

We also analysed the intake of 21 key food groups of the ‘Western’ and ‘Healthy’ dietary patterns in association with mathematics, reading and writing scores in the fully adjusted model. (This is equivalent to ‘model three’ in the previously described analyses, except dietary patterns were not adjusted for). We found that higher intake of confectionery and soft drink were associated with lower scores in mathematics (confectionary: (β = −0.182; 95% CI: −0.328; −0.035; *p* = 0.015; soft drink: (β = −0.032; 95% CI: −0.051; −0.012; *p* = 0.001) and reading (confectionary: (β = −0.246; 95% CI: −0.384; −0.108; *p* ≤ 0.001; soft drink: (β = −0.022; 95% CI: −0.041; −0.003; *p* = 0.023). We also found that a higher intake of processed meat (β = −0.307; 95% CI: −0.520; −0.093; *p* = 0.005) and fried potato (β = −0.497; 95% CI: −0.937; −0.058; *p* = 0.027) were associated with lower scores in reading. Higher intake of yellow and red vegetables were associated with higher scores in mathematics (β = 0.292; 95% CI: 0.038; 0.546; *p* = 0.024) and reading (β = 0.284; 95% CI: 0.048; 0.520; *p* = 0.018), while higher intake of fresh fruit was associated with higher scores in mathematics (β = 0.034; 95% CI: 0.001; 0.067; *p* = 0.044). Higher intake of wholegrain was associated with higher scores in reading (β = 0.102; 95% CI: 0.015; 0.188; *p* = 0.022). The small β values reflect 1gram difference in food group intake. None of the specific food groups showed significant associations with writing. Significant results are illustrated in Figure 2.

## 4. Discussion

We examined the associations between adolescents’ dietary pattern intake and academic performance in the Raine cohort at age 14 and found that a higher *z*-score for the ‘Western’ dietary pattern was associated with significantly poorer academic performance (specifically, poorer scores in mathematics, reading and writing) independent of maternal education and race, family income, functioning and structure. These results showed an average of 1SD difference to the means of the academic scores for those children who had the 5th percentile (lowest level) and 95th percentile (highest level) of the ‘Western’ dietary pattern score. The inclusion of adolescent BMI and physical activity level in the analyses did not alter these results. We found positive associations between academic performance and consumption of fruit, yellow and red vegetables and whole grains. Negative associations between academic performance and consumption of confectionary, soft drink, fried potato and processed meat, were observed.

### 4.1. Diet and Academic Performance during Adolescence

Academic performance of adolescents has been shown to be influenced by meal patterns (breakfast, lunch and dinner) and both poor (e.g., consuming French fries, hot dogs, soft drinks), and good (e.g., consuming fruits, vegetables, fish) diets [6,7,8,9,10,28]. These previous reports are consistent with our current findings that show associations between a higher intake of the ‘Western’ dietary pattern and its food components with lower academic performance.

Only one study to date has reported on the positive association between whole diet quality and academic performance in children in grade five [29]. In another study undertaken by our research group in the same cohort, the ‘Western’ dietary pattern at age 14 had a negative influence on 17 year old adolescents’ cognitive performance, specifically with respect to their psychomotor and executive functioning [30]. It is known that cognitive performance is a significant predictor for academic achievement [31].

We showed in the current study that BMI manifested significant associations with educational attainment in mathematics but not with reading and writing, and that physical activity was not associated with academic performance. However, our results were not modified by including BMI and physical activity in the multivariate regression modelling with academic performance as the outcome. This is in contrast with other studies that have found associations between physical activity, BMI, dietary factors and academic performance in children and adolescents [8,32,33].

### 4.2. Mechanisms Underlying the Association between Diet and Academic Performance during Adolescence

Adolescence is a sensitive developmental time period for the brain, particularly with respect to the prefrontal cortex and other important brain structures, including the hippocampus which is critically involved in learning and memory [34]. It is plausible that diet could be a significant environmental factor influencing brain plasticity during this sensitive time period.

The ‘Western’ dietary pattern (which includes a high intake of ‘take away’ foods, red and processed meat, soft drinks, and fried and refined food) correlates with overall intake of total fat, saturated fat, refined sugar and sodium [15]. Moreover, this ‘Western’ dietary pattern has been associated with increased biomarkers predictive of the metabolic syndrome in the same cohort [35]. Further, high fat and refined carbohydrate consumption and metabolic syndrome and its biomarkers have all been linked with cognitive dysfunction (and possibly lower academic performance) through hippocampal and frontal lobe volume loss and dysfunction [36,37]. This dysfunction may be due to neuroinflammation, oxidative stress, damaged blood-brain barrier and/or abnormal brain lipid metabolism [36,37].

The ‘Western’ dietary pattern is not only high in saturated fat, refined sugar and sodium but is also poor in micronutrients. Micronutrients are necessary for brain function. Specifically, folate has been positively associated with academic achievement in 15 year old adolescents [38], while iron deficiency has been linked to poorer mathematics scores in both children and adolescents [39]. Conversely, associations between higher intake of fruit and yellow red vegetables and better mathematics and reading scores may be due to increased levels of micronutrient content in these foods.

Another possible explanation for our findings is that adolescents who scored higher on the ‘Western’ dietary pattern may have been less likely to eat breakfast regularly. Children who regularly eat breakfast tend to have better overall diet quality [40] (and possibly *vice versa*). In another study in the Raine cohort, a positive correlation was observed between breakfast quality and overall diet quality [41]. A good quality (*i.e.*, low glycaemic index) breakfast has been linked to better academic performance, while poor quality breakfast or no breakfast has been linked to poorer academic scores in children and adolescents [6,40,42]. Breakfast provides the brain with fuel (glucose) after an overnight fast, which is important in preserving brain functions. In addition, those children who eat breakfast generally have higher micronutrient intake compared to those who skip breakfast [42].

### 4.3. Strengths and Limitations

One of the strengths of our study is that academic performance scores including mathematics, reading and writing were uniformly obtained by an independent organization via national testing. Further, we were able to adjust for a range of maternal and family socioeconomic covariates and adolescent characteristics that may have represented confounding.

A limitation of our study was that, since this was a cross- sectional analysis, we are not able to claim cause and effect in the observed relationships. Another limitation is that we were not able to adjust for maternal intelligence. However, we were able to adjust for maternal education, which is a valid proxy measure for maternal intelligence [43]. Finally, we acknowledge that we cannot rule out the possibility of other confounding factors that we were not able to adjust for in our analyses and which may have been significant drivers of academic performance.

## 5. Conclusions

We have identified a ‘Western’ dietary pattern as a risk factor for poorer academic performance during adolescence. Adolescence is a sensitive period for brain development and a vulnerable time of life with respect to nutrition. Therefore, public health policies and health promotion programs should rigorously target the issue of food intake during this stage of individual development. To date, this is one of the few studies to report on the associations between dietary patterns and academic performance; therefore, more prospective studies are required to support our findings.

## Supplementary Files

Supplementary File 1
