# Supplementary material for: A Western Dietary Pattern Is Associated with Poor Academic Performance in Australian Adolescents
Source: Nutrients. 2015 Apr 17;7(4):2961–82. doi: 10.3390/nu7042961 (PMC4425183; doi:10.3390/nu7042961)
Supplement: Supplementary File 1 [file nutrients-07-02961-s001.docx]

**Supplementary Information**

**Table S1.** Dietary patterns and their factor loadings in the Western Australian Pregnancy (Raine) Cohort at 14 years [1] *.

| **Food Group** | **Factor Loadings** | |
| --- | --- | --- |
|  | **‘Healthy’ Pattern** | **‘Western’ Pattern** |
| Takeaway foods | −0.20 | 0.53 |
| Confectionery | −0.14 | 0.46 |
| Red meat | 0.14 | 0.46 |
| Refined grains | 0.03 | 0.42 |
| Processed meats | −0.02 | 0.41 |
| Potato, fried e.g., french fries | −0.25 | 0.39 |
| Crisps (potato chips) | −0.22 | 0.39 |
| Soft drinks | −0.18 | 0.37 |
| Cakes, biscuits | 0.10 | 0.34 |
| Potato, not fried | 0.21 | 0.34 |
| Sauces and dressings | 0.13 | 0.34 |
| Full fat dairy products | 0.00 | 0.30 |
| Yellow, red veg | 0.56 | 0.12 |
| Leafy green veg | 0.49 | 0.00 |
| Tomato | 0.49 | 0.00 |
| Cruciferous veg | 0.48 | 0.27 |
| Other vegetables | 0.66 | 0.22 |
| Fresh fruit | 0.48 | −0.02 |
| Legumes | 0.43 | 0.19 |
| Wholegrains | 0.39 | −0.12 |
| Fish, steamed, grilled or tinned | 0.33 | 0.05 |
| Poultry | 0.01 | 0.29 |
| Soups | 0.26 | 0.26 |
| Eggs | 0.20 | 0.24 |
| Fish, fried or battered | 0.02 | 0.23 |
| Added sugar | 0.13 | 0.21 |
| Milk dishes | 0.13 | 0.20 |
| Meat dishes | 0.26 | 0.15 |
| Canned fruit | 0.26 | 0.11 |
| Dried fruit | 0.23 | 0.00 |
| Juices | 0.19 | −0.02 |
| Nuts | 0.17 | −0.02 |
| Mineral water | 0.23 | −0.05 |
| Low fat dairy products | 0.22 | −0.10 |
| *Variance* | *4.28* | *2.89* |
| *% common variance* | *50* | *34* |
| *Mean* | 0 | 0 |
| *Standard deviation* | 0.89 | 0.87 |
| *Min* | −2.12 | −2.07 |
| *Median* | −0.1 | −0.13 |
| *Max* | 5.01 | 4.74 |

Foods having a factor loading of ≥30 are highlighted in bold. * Table reproduced here from Ambrosini *et al.* [1].

**Reference**

1. Ambrosini, G.L.; Oddy, W.H.; Robinson, M.; O’Sullivan, T.A.; Hands, B.P.; de Klerk, N.H.; Silburn, S.R.; Zubrick, S.R.; Kendall, G.E.; Stanley, F.J.; *et al.* Adolescent dietary patterns are associated with lifestyle and family psycho-social factors. *Public Health Nutr.* **2009**, *12*, 1807–1815.

© 2015 by the authors; licensee MDPI, Basel, Switzerland. This article is an open access article distributed under the terms and conditions of the Creative Commons Attribution license (http://creativecommons.org/licenses/by/4.0/).
